# Editorial: Autophagy in diseases—From basic to clinic

**DOI:** 10.3389/fphys.2022.1115511

**Published:** 2023-01-04

**Authors:** Jie Yang, Zhenhong Ni, Huifeng Pi, Adam Bohnert, Zhiqiang Deng

**Affiliations:** ^1^ Institute of Cardiovascular Diseases of PLA, The Second Affiliated Hospital, Third Military Medical University (Army Medical University), Chongqing, China; ^2^ Department of Cardiology, The Second Affiliated Hospital, Third Military Medical University (Army Medical University), Chongqing, China; ^3^ State Key Laboratory of Trauma, Burns and Combined Injury, Department of Rehabilitation Medicine, Daping Hospital, Army Medical University, Chongqing, China; ^4^ Institute of Military Preventive Medicine, Army Medical Univeristy, Chongqing, China; ^5^ Louisiana State University, Baton Rouge, LA, United States; ^6^ School of Chinese Medicine, Mr. and Mrs. Ko Chi Ming Centre for Parkinson's Disease Research, Hong Kong Baptist University, Kowloon, Hong Kong SAR, China

**Keywords:** autophagy, autophagy-related diseases, autophagy regulation, autophagy-related clinical practice, diabetes, cardiovascular diseases, protozoa

Autophagy is an evolutionarily conserved, lysosome-dependent catabolic process during which cytoplasmic components, including damaged organelles, protein aggregates, and lipid droplets, are degraded and recycled. It plays an essential role in the maintenance of cellular homeostasis in response to intracellular stress. The efficiency of autophagy is altered with age, and numerous diseases can also interfere with this process. Autophagy insufficiency or over-activation is confirmedly associated with numerous human diseases, such as stem cell regeneration, hypoxia-related diseases, cardiovascular diseases, aging, and metabolic disorders. However, its role and mechanism of action in disease regulation are still under debate.

This Research Topic provides a Research Topic of four articles (three review articles and one original research article) that deepen and develop our understanding of how autophagy is involved in disease regulation and treatment. Each article illustrated the various connections between autophagy and common diseases, including arrhythmia, diabetes, chronic kidney disease, and parasitic diseases.

The original article (Front Physiol. 2022; 13: 960480) highlighted the role of autophagy in the intracardiac thrombosis process of atrial fibrillation (AF), the most common tachyarrhythmia in the world. This study revealed that the change in hemodynamics in the left atrium of the AF model resulted in autophagy inhibition in the endocardial endothelial cells (EEC). Sun et al. demonstrated that EECs from AF mice exhibited increases in expression of plexin D1 (PLXND1) and accumulation of PLXND1 in the plasma membranes. PLXND1 is a mechanosensor for fluid shear, and its expression level is implicated in the development of atherosclerosis (Mehta et al., 2020). Besides, in this study PLXND1 was found to be a scaffold protein that binded with the calcium channel protein ORAI1. Free ORAI1 resulted in a decrease in calcium influx, CAMK1 phosphorylation, and autophagy inhibition, during cell membrane reduction. This study proposes a new autophagy regulation field in which hemodynamics regulate autophagy flux through changes in the blood-flow shear stress. In addition, this study also provides a potential intervention target for EEC dysfunction, to prevent and treat intracardiac thrombosis in AF and its complications.

The review article from Ge et al. (Front Physiol. 2022 August 8;13:956344) focused on mitophagy in cardiorenal syndrome (CRS). Mitochondrial injury is a common pathological change in both the kidney and heart in CRS patients. Mitophagy preserves mitochondrial homeostasis and provides quality control by selectively eliminating the damaged mitochondria (Klionsky et al., 2021). Therefore, targeting mitophagy might ameliorate both chronic kidney disease (CKD) and cardiovascular disease (CVD), which sheds new light on its role as a promising therapeutic target for CRS. Based on this hypothesis, this review elaborates on the potential drugs that regulate mitophagy in CRS treatment, including statins (Yang et al., 2022), ACEI (Zhang et al., 2014), and metformin (Zhao and Sun, 2020). In addition, Yao et al. (Front Physiol. 2022 October 24;13:1008517) suggested that stem cell transplantation has therapeutic effects in CRS, but due to the complex source and poor quality control of stem cells, the safety and effectiveness of stem cell therapy are still under debate (Sivanathan and Coates, 2020). Therefore, stem cell exosomes, containing small molecular mitophagy activators, such as UMI-77 (Jin et al., 2022) and α-ketoglutarate (An et al., 2021), may be a replacement and emerge as a promising cell-free therapy for CRS. Similarly, Ge et al. (Front Physiol. 2022 August 8;13:956344) systematically examined the molecular mechanisms of autophagy in the chronic complications of diabetes, including retinopathy, nephropathy, and cardiomyopathy with detailed drawings. Notably, this work describes and summarizes the latest autophagy-related drugs to improve diabetes and its complications, including the glucagon-like peptide 1 (GLP-1) receptor agonist liraglutide (Xue et al., 2019), GLP-1 receptor agonist exendin-4 (Al-Bari and Xu, 2020), pioglitazone (Xu et al., 2020), SGLT-2 inhibitor empagliflozin (Packer, 2020), and DPP-4 inhibitor linagliptin (Korbut et al., 2020). These results highlight the role of autophagy in the amelioration of diabetic complications and contribute to the research on and application of glucose-lowering drugs related to the molecular mechanisms of autophagy. Currently, common approaches for modulating autophagy in diabetes include the use of autophagy inhibitors or knockout of autophagy-related genes, which have shown good modulation in animal experiments but still lack clinical trials. Therefore, further research is required on drugs that modulate autophagy, which may be a potential research direction for the treatment of chronic complications related to diabetes.

Autophagy not only exists in mammalian cells but has also been observed in protozoa since 1977, based on ultrastructural evidence. Parasites under starvation or subjected to other stress conditions commonly present with autophagic features, including an increase in the number of autophagosomes, multivesicular bodies, and myelin-like structures. In this Research Topic, Pedra-Rezende et al. (Front Microbiol. 2022 March 29;13:856686) introduced different molecular mechanisms of autophagy in protozoa and listed the main protozoal infections and their current chemotherapies. Interestingly, this study exhibited autophagic phenotypes in the different protozoa (Trypanosoma cruzi, Trichomonas vaginalis, and Leishmania braziliensis), using transmission electron microscopy, during the drug treatment. These autophagic phenotypes represent the survival machinery responsible for the removal of cellular structures that have been damaged by compounds that may play a positive role in the drug resistance and susceptibility of these pathogens.

Although, this Research Topic has been successfully published, there is still several limitations. First, the diseases and cell types involved are limited. Autophagy has been considered to be associated with nearly all pathological process, especially in neurodegenerative diseases. Unfortunately, there is no review or original article refers to neurodegenerative diseases. Second, autophagy detection method and intervention technology have not yet been discussed in this Research Topic. We believe that with the deepening of autophagy research, new autophagy-associated molecules and mechanisms will be gradually revealed. In addition, at present, the autophagy flux detection method is limited, new techonology of autophagy flux evaluation is expected to explore and to apply in near future.

In summary, the articles that were studied in this Research Topic focused on autophagy in common diseases, which helps the readers understand the basic autophagy changes during disease progression and the effect of different treatment agents on autophagy flux. Although only a few articles were included in this Research Topic, they ranged from mammalian to protozoa and from cardiovascular diseases to infectious diseases and provided a broad overview of the treatments targeting autophagy. Undoubtedly, there is still a considerable amount of work required to explore autophagic targets in different diseases, apply autophagy regulation in clinical practice, and benefit patients.
